# Multifunctional dynamic cerium-polypeptide hydrogel with antibacterial antioxidative anti-inflammatory for multidrug-resistant bacterial infected wound healing

**DOI:** 10.1093/rb/rbaf071

**Published:** 2025-07-01

**Authors:** Meng Luo, Jing Tian, Chenxi Xie, Yanzi Zhao, Bo Lei

**Affiliations:** School of Life Science and Technology, Xidian University, Xi’an 710126, PR China; Frontier Institute of Science and Technology, Xi’an Jiaotong University, Xi’an 710054, PR China; Frontier Institute of Science and Technology, Xi’an Jiaotong University, Xi’an 710054, PR China; Frontier Institute of Science and Technology, Xi’an Jiaotong University, Xi’an 710054, PR China; Frontier Institute of Science and Technology, Xi’an Jiaotong University, Xi’an 710054, PR China; Frontier Institute of Science and Technology, Xi’an Jiaotong University, Xi’an 710054, PR China

**Keywords:** bioactive biomaterials, dynamic hydrogel, antibacterial, infected wound

## Abstract

Bacterial infection, especially multidrug-resistant (MDR) bacterial infection, is a great challenge in clinical wound repair, highlighting the urgent to develop antibacterial hydrogel dressing. In this work, a multifunctional cerium-polypeptide hydrogel (FEPC) with comprehensive antibacterial, antioxidant, anti-inflammatory and angiogenesis ability was developed for the treatment of methicillin-resistant *Staphylococcus aureus* (MRSA) infected wounds. The FEPC hydrogel was constructed using Ce^3+^ and antibacterial polypeptide co-crosslinked γ-polyglutamic acid (γ-PGA) through double dynamic electrostatic and coordination interaction. This unique dynamic architecture endowed FEPC with outstanding injectability and rapid self-healing capacity, ensuring conformal coverage of irregular wounds. *In vitro* experiments demonstrated that FEPC showed robust antibacterial activity, and could effectively eliminate reactive oxygen species (fixed terminology). Meanwhile, the FEPC hydrogel could downregulate the expression of pro-inflammatory cytokines and promote angiogenesis by upregulating the VEGF expression. Importantly, *in vivo* assessments using MRSA-infected full-thickness wounds showed that FEPC hydrogel could rapidly repair the MRSA infected wounds (54% wound repair rate on Day 3 for FEPC *vs* reported hydrogel below 40% rate) and promote epithelialization and hair follicle regeneration within 14 days. Histological analysis confirmed that FEPC hydrogel could significantly inhibit infection and excessive inflammation, as well as accelerate angiogenesis. This work suggests that cerium-polypeptide hydrogel is a perfect partner for treating MDR bacterial infected wounds, providing a viable solution for synergistically combat infection, oxidative stress and other related-disease treatments.

## Graphical abstract

**Figure:**
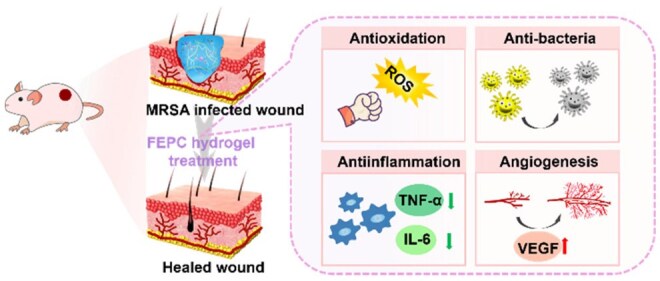

## Introduction

Skin is the largest organ of the human body and has an important protective barrier function [1]. Once the skin is damaged, it is easy to develop wound infection, aggravate the skin damage and even lead to the death of the patients [2]. Wound healing has always been a global public health problem, as well as the focus of medical research, which has brought serious challenges and economic burden to society [3, 4]. Wound healing is a complex and interacting physiological process, including four stages: hemostasis, inflammation, proliferation and remodeling [5]. Bacterial infection is one of the most important causes of slow healing of chronic wounds, because severely infected wound beds can accumulate large amounts of reactive oxygen species (ROS), causing excessive inflammation, which may delay the healing time and even lead to the risk of death [6–8]. Traditionally, gauze, bandages, cotton, etc. can provide physical protection for damaged wounds, but they are prone to cause secondary injury, which also are poorly matched to the wound and have weak bioactive functions. Clinically, antibiotics are often used to kill bacteria and prevent infection [9]. However, the overuse of antibiotics will lead to the increase of multidrug-resistant (MDR) bacteria, which makes it more difficult to treat bacterial infections and seriously affects the process of wound healing [10–12]. Zhao *et al*. have designed a photocatalytic and antibacterial MOF nanozyme for infected wound repair [13]. Therefore, there is an urgent need to construct novel platforms to target MDR bacterial infections and promote wound healing and tissue regeneration.

Hydrogels are widely used in tissue repair due to their three-dimensional (3D) porous structure and extracellular matrix (ECM)-like properties, which can maintain a moist environment and absorb tissue exudates [14, 15]. However, traditional covalently crosslinked hydrogels face some problems in practical applications [16]. For example, the hydrogel dressings may detachment or be torn owing to frequent body movement, or neither fully fill the irregular wounds. Notably, dynamic hydrogels with break-recombination ability are more suitable for wound repair. Dynamic hydrogels can restore their original structure and function after injury due to the reversibility of their dynamic bonds [17]. At present, some typical dynamic cross-linked bonds have been developed, including Schiff base reactions, hydrogen bonds, boronate ester bonds, host–guest interactions, π–π stacking, hydrophobic interactions, electrostatic interaction and coordination interactions [18–20]. Our group has developed a series of dynamic hydrogels through hydrogen bonding and coordination interactions that exhibited excellent injectable and self-healing properties for acute wound healing [21, 22]. Given that exposed wound is susceptible to bacterial infection, especially MDR bacteria, hydrogels for the treatment of MDR bacterial-infected wounds should be multifunctional and proactive.

γ-Polyglutamic acid (γ-PGA), an anionic polypeptide, is a natural biological macromolecule composed of glutamic acid residues linked by γ-carboxyl groups, which can interact with amino groups, hydroxyl groups and sulfhydryl groups on the surface of tissues [23, 24]. γ-PGA has good water solubility, biocompatibility and biodegradability due to its similarity to ECM structure [25]. In addition, the abundant free carboxyl groups on the side chain of γ-PGA enable it to form dynamic bonds with various polymer molecules, which has been widely used in agriculture, pharmaceuticals, cosmetics, drug delivery and tissue engineering [26–28]. ε-polylysine (EPL) is a natural cationic polypeptide, which is widely used in food preservative and biomedical fields due to its good biocompatibility and broad-spectrum antibacterial properties [29, 30]. The surface of EPL has a large amount of positive charge, which can attach to the negatively charged bacterial membrane and eventually destroy the shape and integrity of the bacterial membrane [31]. In addition, EPL possess good water solubility, biodegradability and no toxicity [32]. Notably, EPL is easily grafted onto other polymers and retained excellent antibacterial activity.

**Figure 1.**
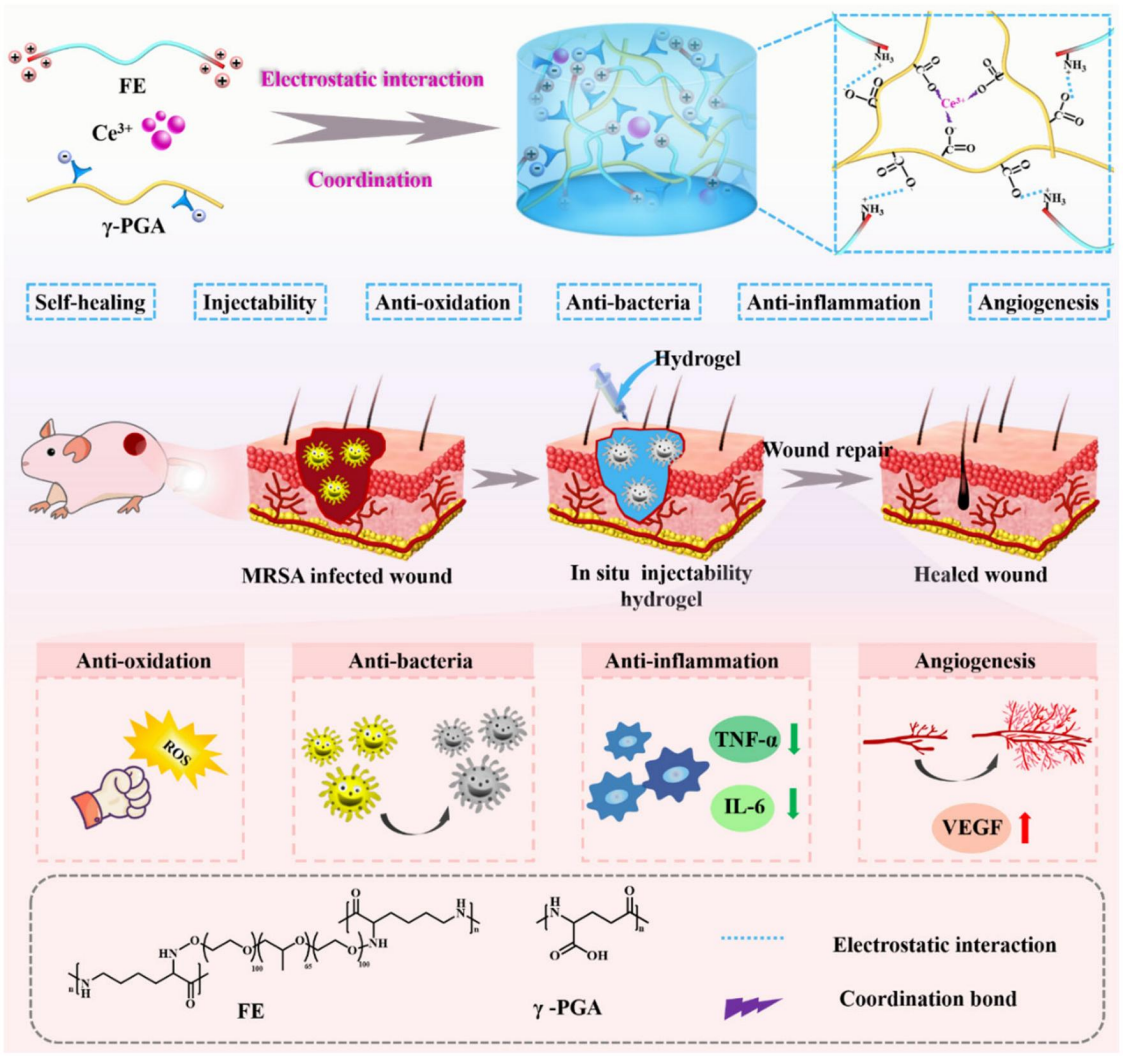
The schematic illustration of synthesis and potential application in MDR bacterial infected wound repair of FEPC hydrogel.

Herein, a multifunctional and adaptable hydrogel (FEPC) was synthesized by dynamic cross-linking *via* electrostatic and coordination interactions for the treatment of MDR bacterial infected wounds (Figure 1). The dynamic hydrogel was composed of γ-PGA, EPL grafted pluronic F127 (FE) and the bioactive ion, Ce^3+^, through the electrostatic interaction between the carboxyl group on γ-PGA and the amino group on FE and the coordination cross-linking between the carboxyl group on γ-PGA and Ce^3+^, which presented satisfactory injectability and self-healing performance to adapt the wounds. The FEPC hydrogel presented good biocompatibility, antioxidation and antibacterial behaviors. Further, *in vivo* study demonstrated that the FEPC hydrogel could significantly promote the MRSA infected wound repair by availably eradicating MRSA bacterial infections, suppressing inflammatory response *via* downregulating the expression of interleukin-6 (IL-6) and tumor necrosis factor-α (TNF-α) as well as promoting angiogenesis and collagen deposition. Collectively, this work developed a neoteric dynamic hydrogel to achieve infected wound therapy.

## Materials and methods

### Synthesis and characterizations of F127-EPL (FE) polymer and FEPC hydrogel

FE polymer was synthesized *via* a two-step synthetic route according to our previous study [33]. Firstly, the intermediate of F127-TsCl was prepared by reaction of F127 and p-toluenesulfonyl chloride. Then, the as-prepared F127-TsCl was reacted with ε-polylysine to obtain the FE polymer. The detailed synthetic process was shown in the Supplementary Material.

The FEP hydrogel was mainly formed by electrostatic interaction between FE and γ-PGA. A certain proportion of FE (25 wt%) and γ-PGA (25 wt%) was mixed under ice bath. Then, the mixture was standing at room temperature for a while to obtain FEP hydrogel. The FEPC hydrogels were synthesized in a similar way to FEP with the addition of different amounts of Ce (0.01M, 0.05M, 0.1M, named as FEPC-0.01, FEPC-0.05, FEPC-0.1).

### Physicochemical properties

The mechanical performance of the hydrogels was measured using a TA rheometer (DHR-2). The injectability and self-healing properties were evaluated according to our previous study. The degradation behavior was also tested *in vitro* using phosphate buffer saline (PBS). The antioxidant behavior was measured using the ABTS assay kit. The specific experimental methods were described in the Supplementary Material.

### Hemocompatibility and cytocompatibility evaluation

The mouse red blood cells (RBCs) were used to test the blood compatibility of the hydrogels based on our previous study, and the operation method was presented in detail in the Supplementary Material [18]. The cytotoxicity was evaluated using the hydrogel extract on the murine fibroblast cell line (L929 cells), human umbilical vein endothelial cells (HUVECs) and macrophage cells (RAW264.7). Cell culture and specific experimental procedures were described in detail in Supplementary Material.

### Cell migration, pro-angiogenesis and anti-inflammation evaluation

The effect of the FEPC hydrogel on cell migration was estimated using HUVECs. Subsequently, the pro-angiogenesis of the FEPC hydrogel was also evaluated by HUVECs. The expression of the vascular endothelial growth factor (*VEGF*) was used to evaluate the pro-angiogenesis ability. The LPS-induced RAW264.7 was used to assay the anti-inflammatory capacity of the FEPC hydrogel. The expression levels of interleukin-6 (*IL-6*) and tumor necrosis factor-α (*TNF-α*) were applied to assess the anti-inflammatory behavior of the FEPC hydrogels. The specific experimental procedures were presented in Supplementary Material.

### Antibacterial ability evaluation

The plate counting method was applied to evaluate the antibacterial performance *in vitro*. The *Escherichia coli* (*E. coli*, Gram-negative bacteria), *Staphylococcus aureus* (*S. aureus*, Gram-positive bacteria) and methicillin-resistant *Staphylococcus aureus* (MRSA, multidrug resistant bacteria) were used to estimate the antibacterial performance of FEPC hydrogels *in vitro*. The detailed process was described in the Supplementary Material.

### MRSA infected wound healing evaluation

All animal experiments procedures were performed in accordance with the protocols authorized by the animal care committee of Xi’an Jiaotong University (No. XJTULAC201). The effect of the FEPC hydrogel on MRSA infected wound repair was examined using a full-thickness MRSA infected skin wound defect as a model according to our previous study (*n* = 6). The wounds were monitored using the camera and the Hematoxylin-Eosin (H&E), Masson’s trichrome-staining and immunofluorescence staining were used to analyze the wound tissue. The detailed experimental process was shown in the Supplementary Material.

### Statistical analysis

All data in this article were showed as mean ± standard deviation (SD). The Student’s *t*-test was employed to calculate the significance of differences between two different groups, and statistical significance among multiple groups was analyzed using one-way or two-way analysis of variance (ANOVA). The results of significant differences were as follows: *P < 0.05 and **P < 0.01.

## Results and discussion

### Synthesis and characterization of FE polymer and FEPC hydrogel

**Figure 2.**
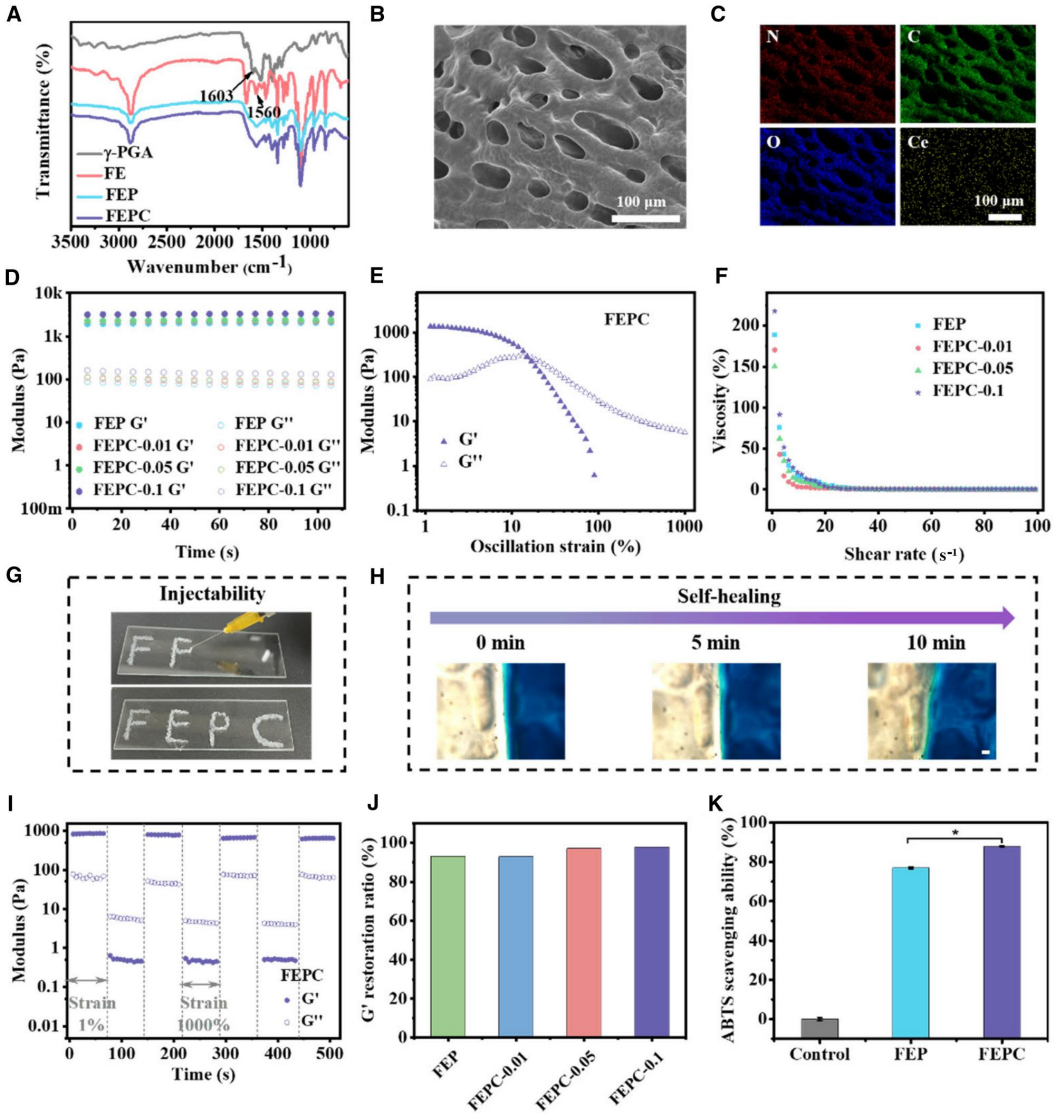
The characterization and multifunction properties evaluation of FEPC hydrogel. (**A**) FTIR spectra; (**B**) SEM image of FEPC hydrogel; (**C**) Elements mapping analysis of FEPC hydrogel; (**D**) The storage modulus (G′) and loss modulus (G″) evaluation of various hydrogels; (**E**) The G′ and G″ of FEPC hydrogel with the oscillation strain changed from 1% to 1000%; (**F**) Viscosity evaluation of the various hydrogel with the shear rate changed from 0.1 to 100 s^−1^; (**G**) Photographs of the injectability of the FEPC hydrogel; (**H**) The self-healing behavior of the FEPC hydrogel, scale bar = 200 μm. (**I**) G′ and G″ of the FEPC hydrogel during three cycles with the step strain between 1% and 1000%; (**J**) The G′ restoration ratio of the various hydrogel after three cycles; (**K**) ABTS free radical scavenging ability of the hydrogels. Data are presented as mean ± SD. Statistical significance was determined using t-test (*n* = 3, *P < 0.05, **P < 0.01).

FE polymer was prepared through a two-step synthesis method (Supplementary Figure S1). The intermediate F127-TsCl and FE polymers were characterized by ^1^H NMR and FTIR spectra. In the ^1^H NMR spectra, the characteristic peaks at 7.86, 7.3 and 2.4 ppm were attributed to the aromatic ring and methyl from TsCl, and the peak at 1.06 ppm was belong to the methyl of F127, suggesting the synthesis of F127-TsCl (Supplementary Figure S2). In addition, the peaks at 3.79, 3.17,1.77, 1.50 and 1.31 ppm were assigned to the methine and methylene groups of EPL, indicating successful formation of FE (Supplementary Figure S3). In the FTIR spectra, the absorption peaks were appeared at 3249 cm^−1^ (-NH-), 1665 cm^−1^ and 1560 cm^−1^ (-C = O-NH-), which was further confirmed that the EPL was successfully grafted to the F127 polymer (Supplementary Figure S4). The FEPC hydrogel was prepared by coordination between Ce^3+^ and carboxyl of γ-PGA and electrostatic interaction between cationic of the FE and anionic of γ-PGA. From the FTIR spectra, the characteristic peak at 1603 cm^−1^ was assigned to -COO- from the γ-PGA (Figure 2A). Once the FE and γ-PGA were mixed together, the electrostatic interaction was immediately occurred. The original peak at 1603 cm^−1^ and 1665 cm^−1^ were disappeared and the peak at 1560 cm^−1^ was broadened (Figure 2A). The as-prepared FEPC hydrogels showed a typical three-dimensional (3D) porous structure (Figure 2B). The composition of the FEPC hydrogel was further validated by element mapping, which showed the N, C, O and Ce elements (Figure 2C).

### Multifunctional performance evaluation

The rheological tests were employed to evaluate the mechanical properties, injectable and self-healing features of the FEPC hydrogel. All hydrogels exhibited higher storage modulus (G′) than loss modulus (G″), suggesting the triumphantly formation the gel at the room temperature. It was worth noting that the FEPC-0.1 hydrogel possesses the highest G′ (3260 Pa) compared with the FEPC-0.01 and FEPC-0.05 hydrogels, indicating that Ce^3+^ could regulate the modulus of the FEP hydrogel (Figure 2D). The dynamic stress sweep tests showed that the G′ reduced and eventually became lower than the G″, when the strain increased, suggesting that the network of the hydrogel was damaged (Figure 2E). The viscosity of the hydrogels dramatically decreased with the shear rate changed from 0.1 to 100 1/s (Figure 2F), demonstrating its injectability. To visually verify the injectable capacity of the FEPC hydrogel, the FEPC hydrogel was extruded from the needle without any hindrance to from an ‘FEPC’ pattern, suggesting its excellent injectability (Figure 2G). In addition, the self-healing behavior of the FEPC hydrogel was estimated by microscopy (Figure 2H). After 10 min, the gap between two pieces of hydrogel was disappeared, indicating the excellent self-healing capacity. The self-healing capability of FEPC hydrogel was further evaluated *via* measuring the G′ and G″ at high (1000%) and low (1%) strains (Figure 2I). The G′ was higher than G″ under low strain (1%), whereas, the G′ was lower than G″ at high strain (1000%). After three cycles, the G′ and G″ were recovered to the original value at the low strain, and the G′ restoration ratio of the various hydrogel was more than 90%, confirming the excellent self-healing capacity (Figure 2J). The self-healing behavior of FEPC was dominated by the electrostatic interaction between FE and γ-PGA, as well as the coordination between Ce^3+^ and γ-PGA. Bioactive materials with injectability and self-healing performance could better facilitate irregular wound repair.

Of note, hydrogel has antioxidant property that is beneficial for wound repair. During the wound healing process, high reactive oxygen species (ROS) levels could cause lipid peroxidation, DNA damage and even generate severe inflammatory environment, leading to difficult wound repair. Therefore, in this study, the 2,2’ - azinobis (3-ethylbenzthiazoline-6-sulfonate) (ABTS) radical assay was employed to measure the overall free radical scavenging capacity of FEPC hydrogel. It was seen that more than 80% of ABTS radical was eliminated after treated with FEPC hydrogel, confirming the striking antioxidant ability (Figure 2K). In addition, the FRAP and DPPH scavenging experiments further confirmed the FEPC hydrogel showed good ROS scavenging capacity (Supplementary Figure S5). Moreover, DCFH-DA probes were utilized to investigate the intracellular ROS scavenging ability of the hydrogels. As shown in Supplementary Figure S6, there was bright green fluorescence in the LPS group, which demonstrated high level of intracellular ROS. Conversely, the green fluorescence was significantly reduced after the FEPC hydrogel treatment, suggesting that the intracellular ROS was effectively removed by the hydrogel. Collectively, the FEPC hydrogel presented excellent multifunctional performance involving injectability, self-healing as well as antioxidant capacity, which could act as a suitable wound dressing for wound repair.

### Biocompatibility evaluation *in vitro*

**Figure 3.**
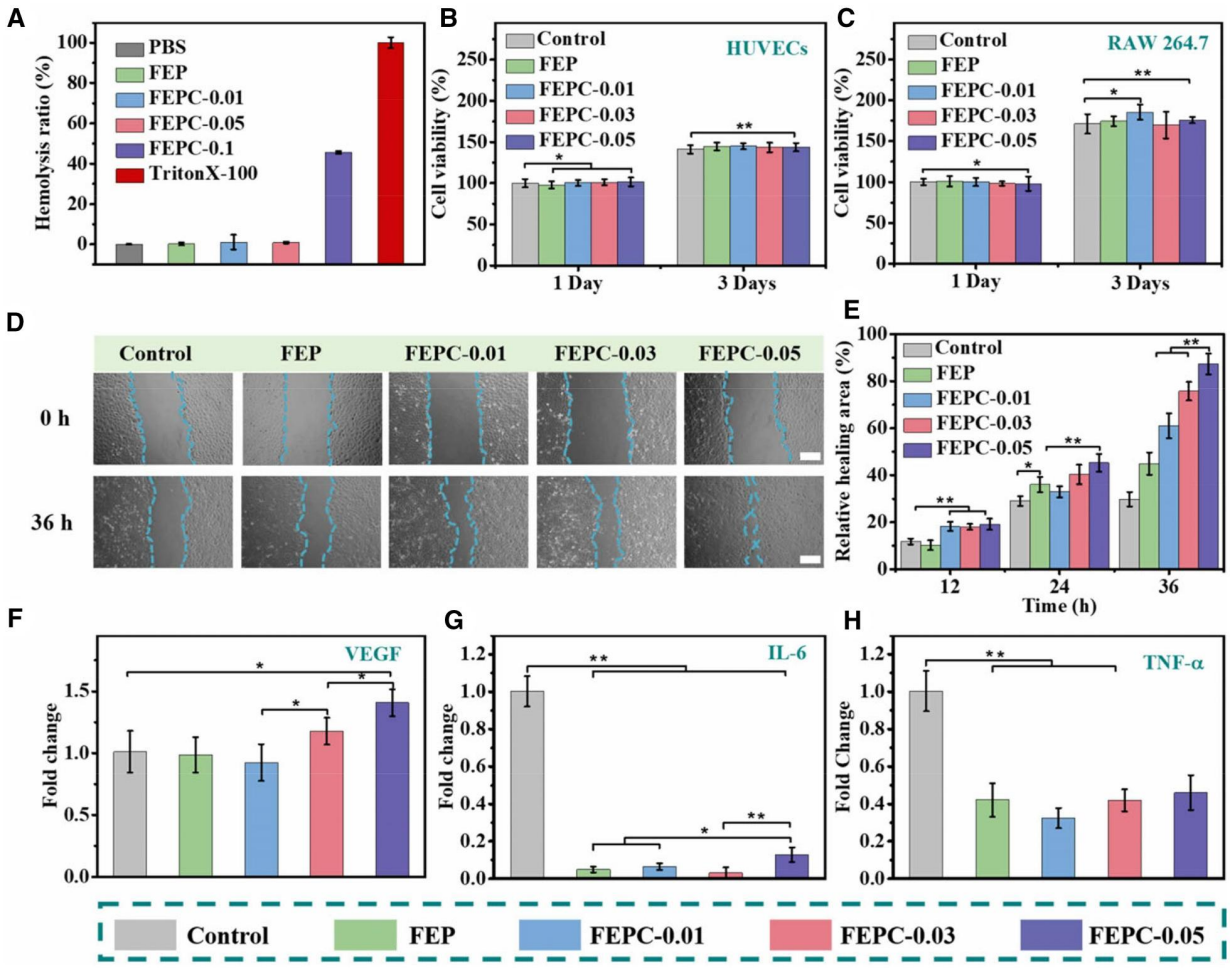
Biocompatibility, cell migration, pro-angiogenesis and anti-inflammation evaluation of the FEPC hydrogel *in vitro*. (**A**) Hemolysis ratio analysis of various groups; (**B**, **C**) cell viability evaluation of the various hydrogels on HUVECs (**B**) and RAW264.7 (**C**); (**D**) Photographs of scratch wounds on 0 hr and 36 hr after treatment of the various groups, scale bar = 200 μm; (**E**) Quantitative result of the relative healing area after treatment of different times (*n* = 3, *P < 0.05, **P < 0.01); (**F**) The mRNA expression of *VEGF* after treated with various hydrogels for 48 hr on HUVECs; (**G**, **H**) The anti-inflammation evaluation of the hydrogels through the mRNA expression analysis of *IL-6* (**G**) and *TNF-α* (**H**). Data are presented as mean ± SD. Statistical significance was determined using ANOVA (*n* = 5, *P < 0.05, **P < 0.01).

An ideal wound dressing should present excellent biocompatibility. Thus, the hemocompatibility and cytocompatibility were employed to evaluate the biocompatibility of FEPC hydrogel. For hemocompatibility estimation, the red blood cells (RBCs) were used to measure the blood compatibility potential. After co-cultured hydrogels and RBCs for 60 min, the FEP, FEPC-0.01 and FEPC-0.05 hydrogels showed a low hemolysis, which was less than 5% (Figure 3A). However, as the concentration of Ce^3+^ was 0.1 mM, the FEPC-0.1 hydrogel exhibited hemolysis phenomenon. Due to the hemolysis of FEPC-0.1 hydrogel, the highest concentration of Ce^3+^ was set to 0.05 mM in the subsequent experiments. In this study, HUVECs and RAW 264.7 cells were used to evaluate the cytocompatibility of the various hydrogels. Compared to the control group, the FEP and FEPC hydrogels exhibited no obvious cytotoxicity to the two cell lines after co-cultured with hydrogels and cells for 3 days (Figure 3B and C). Notably, the HUVECs showed a bit proliferation at Day 3. Taken together, the FEPC hydrogels showed favorable biocompatibility, which were suitable to act as a satisfactory wound dressing *in vivo*

### Cell migration, angiogenesis and anti-inflammatory activities

Cell migration plays a significant role in promoting wound repair. In this work, the impact of FEPC hydrogel on HUVECs migration was evaluated by scratch assay. The HUVECs migration were monitored by the microscope at the various time points of 0 and 36 hr (Figure 3D). The results presented that the HUVECs has migrated after treatment of the various hydrogels compared with the control group. After treated with various hydrogels for 36 hr, the scratch healing rate was 44.94%, 61.2%, 75.72% and 87.34% of the FEP, FEPC-0.01, FEPC-0.03 and FEPC-0.05, respectively, while the healing rate was only 29.8% in the control group (Figure 3E). Of note, the migration rate of HUVECs was the fastest after treated with FEPC-0.05 hydrogel, which almost completely recovered the original scratch wound, which probably was because that Ce^3+^ could promote cell migration and cell proliferation [34]. This result confirmed that FEPC hydrogel possessed outstanding influence on the HUVECs proliferation, which was conducive to the wound repair.

Angiogenesis is essential for nutrition delivery and oxygen penetration, which is contributed to wound repair. In this study, to explore the angiogenic impact of FEPC hydrogels, the expression of vascular endothelial growth factor (VEGF) in HUVECs cultivated with various hydrogels for two days was tested. As presented in Figure 3F, the expression of VEGF was significantly increased after treated with FEPC-0.03 and FEPC-0.05 hydrogel groups compared to the control group. The result was because Ce^3+^ could enhance cell migration, which was a key step of angiogenesis. At the same time, the Ce^3+^ could also induce pro-angiogenesis through stabilizing HIF-1α in the endothelial cells [35]. In addtion, the FEPC hydorgel also could effcteivly enhance the tube formation (Supplementary Figure S7). These results illustrated that FEPC hydrogel could effectively promote angiogenesis that was conducive to wound repair.

Inflammation is the second stage of the wound process, which acts a crucial role in wound repair. The excessive inflammation can delay the healing process. Therefore, to validate the anti-inflammatory effect of the FEPC hydrogel, LPS-stimulated RAW264.7 macrophages were employed as the model *in vitro*. The expression levels of interleukin-6 (*IL-6*) and tumor necrosis factor-α (*TNF-α*) were used to assess the anti-inflammatory behavior of the FEPC hydrogels (Figure 3G and H). After being treated with FEPC hydrogels, the expression of the *IL-6* and *TNF-α* were reduced, exhibiting excellent anti-inflammatory properties. This phenomenon was attributed to that FEPC hydrogel could effectively scavenge ROS, as well as the anti-inflammatory effects of PGA and cerium [36–38], thereby reducing inflammation reaction. Therefore, these results demonstrated that FEPC hydrogel presented good anti-inflammatory behavior and could eliminate the overproduction inflammation in the infected wounds, so that the process of wound healing could smoothly transit from the inflammatory phase to the proliferative phase, thereby promoting wound repair.

### Antibacterial ability evaluation *in vitro*

**Figure 4.**
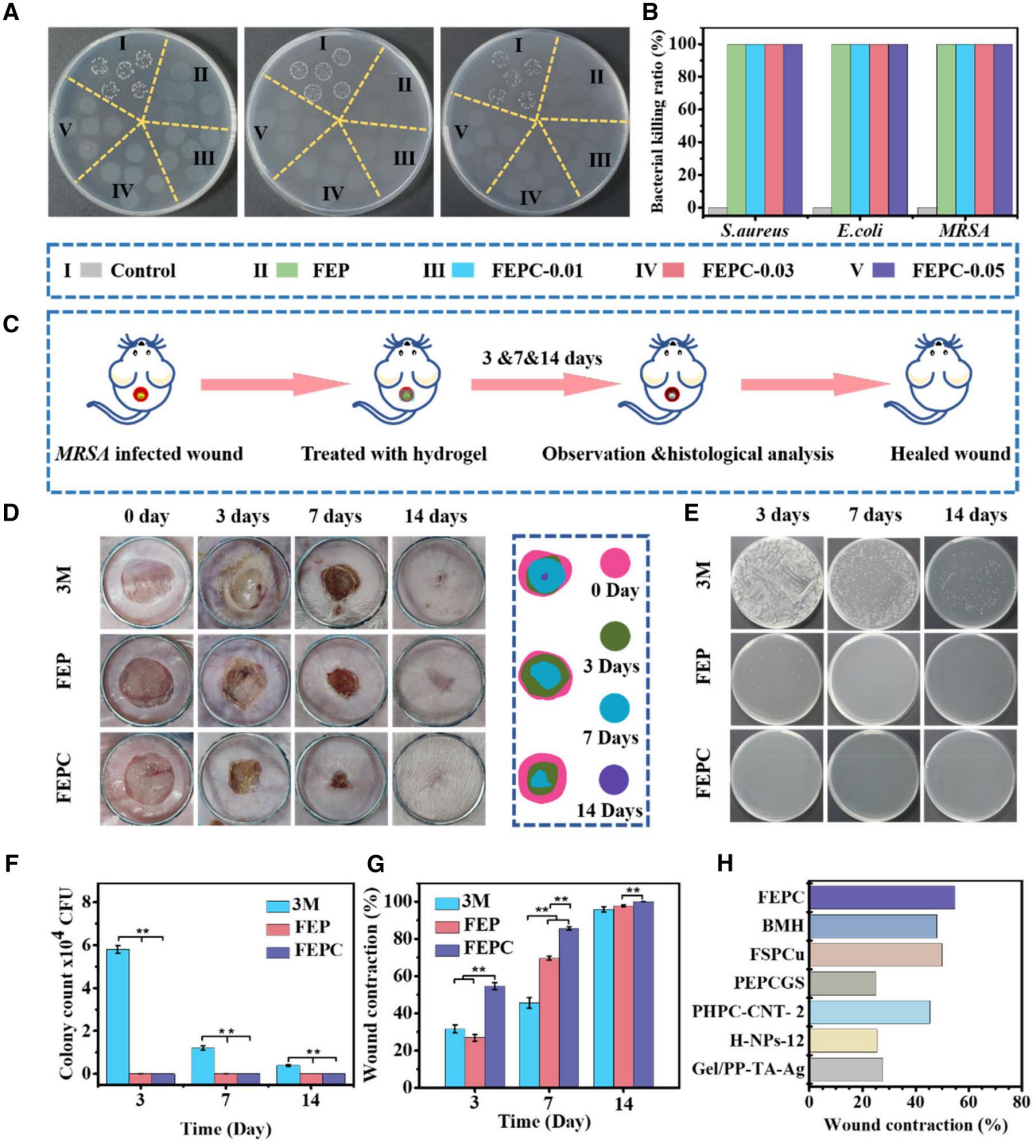
Antibacterial assay *in vitro* and MRSA infected wound repair evaluation *in vivo*. (**A**) The photographs of *S. aureus*, *E. coli* and MRSA colonies growing on LB agar plates after treated with various hydrogel for 2 hr *in vitro*. (**B**) Bacterial killing ratio analysis of the various hydrogel; (**C**) The schematic diagram of the MRSA infected wound repair; (**D**) Representative photographs of wounds treated with 3M, FEP and FEPC hydrogels on Day 0, Day 3, Day 7 and Day 14, scale bar = 5 mm; (**E**) The photographs of MRSA colonies growing on LB agar plates from the wound beds after various treatment; (**F**) Statistics of MRSA colonies after various treatment; (**G**) Wound contraction ratio after treatment of various samples on Day 0, Day 3, Day 7 and Day 14. Data are presented as mean ± SD. Statistical significance was determined using ANOVA. (*n* = 6, *P < 0.05, **P < 0.01); (**H**) Wound contraction on Day 3 with various reported hydrogel for MRSA infected wound.

The skin tissue is severely damaged and easily contaminated by bacteria. Therefore, to protect wound tissue from external bacterial infections, wound dressing should preferably have inherent antibacterial properties. Herein, the antibacterial ability of the FEPC hydrogel was estimated by *E. coli*, *S. aureus* and MRSA. After contacted with the various hydrogels for 2 hr, the three bacteria were effectively killed (Figure 4A). The FEP and FEPC hydrogels showed 100% antibacterial ratio against three kinds of bacteria (Figure 4B). In addition, crystal violet staining was used to evaluate the biofilm scavenging capacity of hydrogels. As shown in Supplementary Figure S8, the inhibition ratio of *S. aureus* biofilm of the FEPC hydrogel was about 86%, suggesting that the hydrogel was able to significantly inhibit the viability of bacteria films. The antibacterial mechanism of FEPC hydrogel was probably that the positive charge on the surface of EPL could combine with the negative charge of bacterial membrane, which could destroy the bacterial structure [39]. Therefore, the above results demonstrated that the hydrogels presented a broad spectrum antibacterial behavior, which could significantly avoid wound infection, indicating a great potential in promoting the healing of MDR bacteria infected wound.

### MRSA-infected wound healing efficiency evaluation *in vivo*

Encouraged by the pronounced antibacterial efficacy *in vitro* of FEPC hydrogel, MRSA infected wounds were constructed to investigate its potential of promoting MDR bacteria infected wound healing *in vivo* (Figure 4C). The process of infected wound healing was monitored by camera. On Day 3, there were a lot of yellow pus in the wound beds after treated with 3M owing to the bacteria infected, suggesting severe inflammation (Figure 4D). However, after treatment of the FEP and FEPC hydrogels, the wounds were scabbed and the area in the FEPC group was the smallest. To validate the anti-infection capacity of FEPC hydrogel, the bacteria were collected from the wound beds and cultured on agar broth plates for 14 hr. It could be observed that there were lots of bacteria after treatment of 3M, while a few bacteria were monitored in the FEP and FEPC hydrogels (Figure 4E), indicating the remarkable antibacterial effect of the hydrogels. The number of the bacteria was further analyzed, which was consistent with the results on the agar broth plates (Figure 4F). This result confirmed that FEPC hydrogel had a striking anti-infection capacity *in vivo*, which could successfully promote the wound repair. As the extension of treatment time, the wound area was obviously reduced in all group on Day 7. The FEPC group had a greater degree of wound contraction with a ratio of 85.6%, while the wound contraction was only 69.7% and 45.57% in the 3M and FEP group (Figure 4G), respectively. What’s more, after treatment with 3M group, the wound was not completely healed even on Day 14. Notably, the wound was healed completely after treatment of FEPC hydrogel with nearly 100% contraction, and the hair was growth on the wound site. Lastly, we compared the wound contraction of reported hydrogels with FEPC hydrogel and found that FEPC hydrogel could significantly accelerate the wound repair (Figure 4H) [40–45]. As well, it took about 15 days to completely repaired in 3M group, while the FEPC hydrogel only spent about 10 days to achieve completely healed (Supplementary Figure S9). Taken together, the FEPC hydrogel could effectively accelerate wound repair due to the superior antibacterial behavior to avoid bacterial infection as well as the natural features of the hydrogel such as exudate adsorption, gaseous exchange and microorganism barrier.

**Figure 5.**
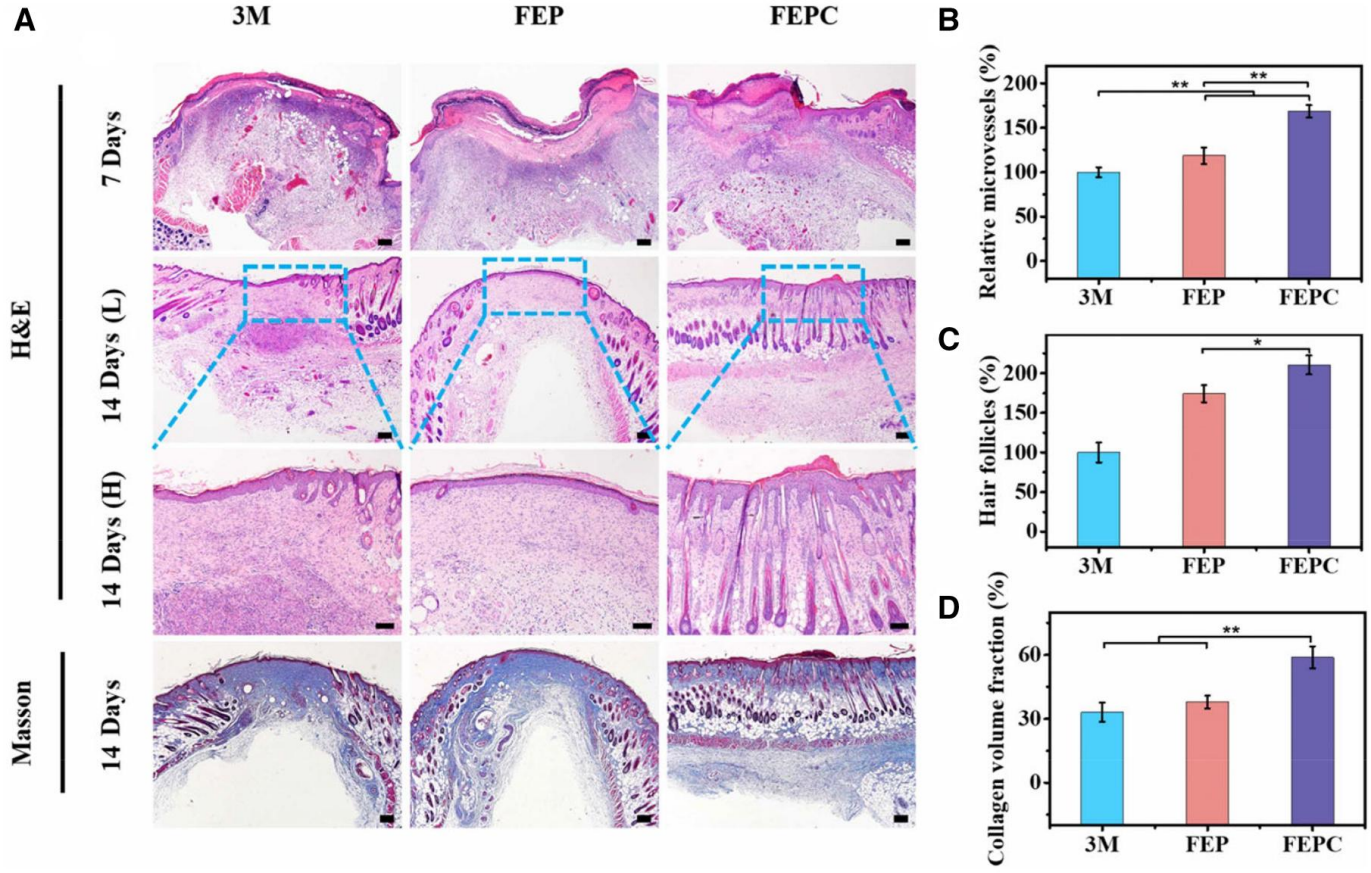
Histological analysis of the MRSA infected wound repair. (**A**) H&E and Masson’s trichrome staining of the wound beds treated with 3M, FEP and FEPC hydrogels on Day 7 and Day 14, scale bar = 200 μm (L), scale bar = 100 μm (H); (**B**) Statistic analysis relative microvessels for various groups on Day 7; (**C**) Hair follicles for various groups on Day 14; (**D**) Collagen volume fraction for various groups on Day 14. Data are presented as mean ± SD. Statistical significance was determined using ANOVA (*P < 0.05, **P < 0.01).

Furthermore, the detailed wound healing process was estimated by the histological analysis. Hematoxylin Eosin (H&E) staining was employed to assess the wound recover situation after various treatments. On Day 3, no epidermal layer was formed after treatment of 3M (Figure 5A). In contrast, the epidermal layers began to form treated with hydrogels. Remarkably, on Day 7, many new blood vessels were formed after FEPC hydrogel treatment (Figure 5B). New blood vessels can provide nutrition to cells in the new skin tissue to maintain the tissue growth, and is necessary for wound repair. In addition, after treated with FEPC hydrogel on Day 14, the skin structure was completely generated accompanied with the abundant hair follicles and sebaceous glands (Figure 5C), which confirmed the best skin regeneration effect. However, there were a few skin appendages after treated with 3M for 14 days. Collagen fibers are a crucial component of the granulation tissues, which play a significant role in wound repair for cell differentiation and tissue repair. Hence, Masson’s trichrome staining was also used to evaluate newly deposited collagen in the wound beds. On Day 14, compared with other groups, the wounds treated with FEPC hydrogel presented more collagen deposition and the collagen fibers were regularly arranged and densely distributed (Figure 5D). Additionally, FEPC hydrogel treated wounds exhibited more skin appendages, consistent with the results of H&E staining. Taken together, these results confirmed that the FEPC hydrogel could effectively accelerate the skin regeneration by eradicating bacteria, promoting angiogenesis and collagen deposition during the wound repair. Considering the long-term biosafety of the FEPC hydrogel, the hydrogel was implanted subcutaneously to evaluate the *in vivo* biocompatibility using histological analysis of the main organs at the specific time point. As shown in Supplementary Figure S10, no significant pathological changes were observed in heart, liver, spleen, lung and kidney, indicating that the FEPC hydrogel could be used a satisfactory biomaterial for wound repair.

### Immunofluorescence staining analysis

**Figure 6.**
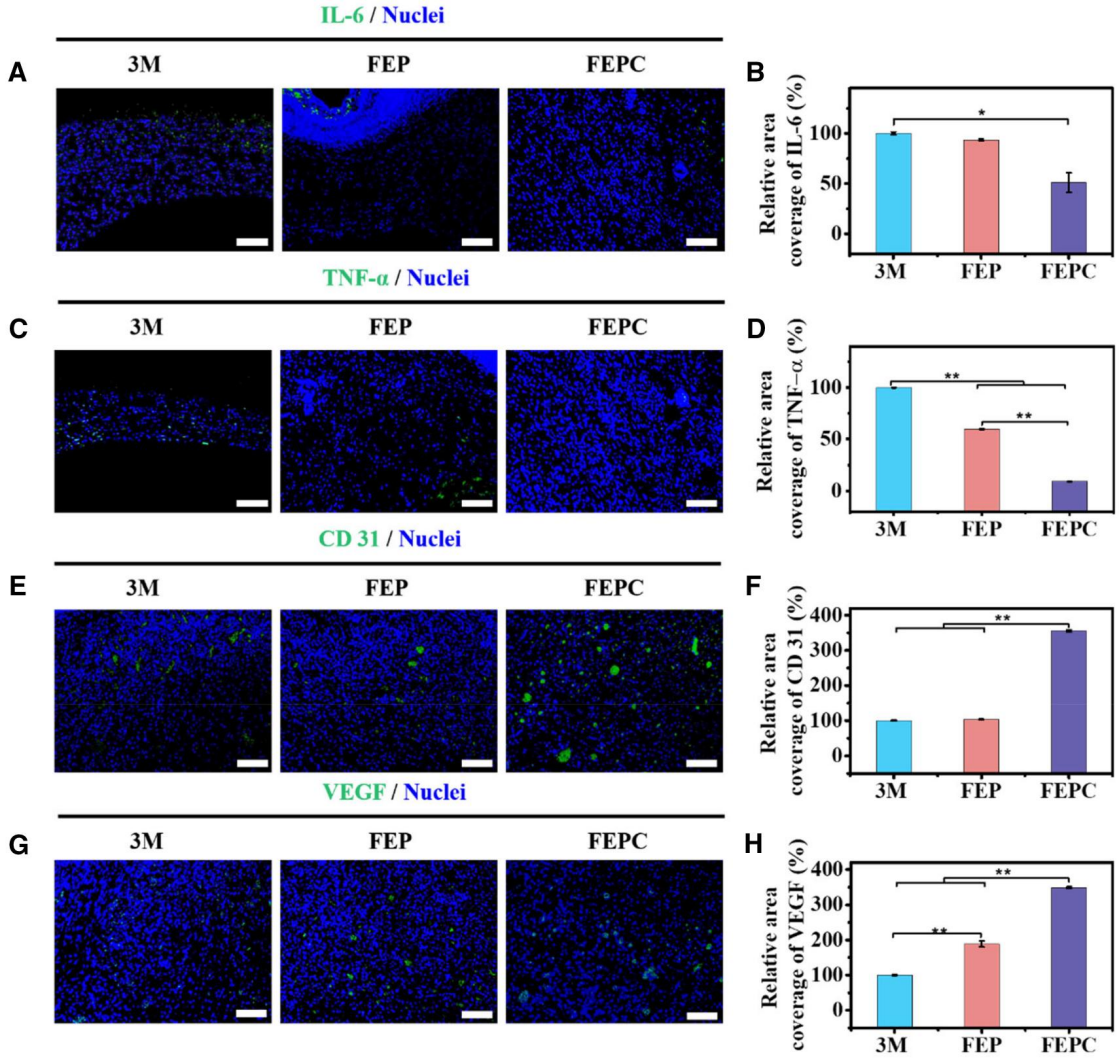
Immunofluorescence staining analysis on inflammation and angiogenesis-related proteins in wound tissue. (**A**) IL-6 immunofluorescence images of wound beds on Day 3, scale bar = 100 μm; (**B**) Corresponding quantification analysis of relative fluorescence intensity of IL-6 in wound beds based on (a); (**C**) TNF-α immunofluorescence images of wound beds on Day 3, scale bar = 100 μm; (**D**) Corresponding quantification analysis of relative fluorescence intensity of TNF-α in wound beds based on (**C**); (**E**) CD31 immunofluorescence images of wound beds on Day 7, scale bar = 100 μm; (**F**) Corresponding quantification of relative fluorescence intensity of CD31 at the wound beds based on (**E**). (**G**) VEGF immunofluorescence images of wound beds on Day 7, scale bar = 100 μm; (**H**) Corresponding quantification of relative fluorescence intensity of VEGF at the wound beds based on (**G**). Data are presented as mean ± SD. Statistical significance was determined using ANOVA. (*P < 0.05, **P < 0.01).

It has been reported that bacterial infection could cause severe inflammatory response that was detrimental to wound repair and delay the wound healing process [46]. Here, interleukin-6 (IL-6) and tumor necrosis factor-α (TNF-α) were employed to assess the antibacterial and anti-inflammatory effects of the hydrogels (Figure 6). There were a large number of positive signals of the IL-6 in the 3M group, while the IL-6 level was reduced in the FEPC groups on Day 3 (Figure 6A and B). Similarly, in FEPC group, the level of TNF-α was decreased, indicating the anti-inflammatory behavior (Figure 6C). However, there were more positive TNF-α in the FEP and 3M groups, implying a severe inflammatory activity (Figure 6D), which was because the FEPC hydrogel exhibited superior antibacterial activity and could abate inflammation. In addition, the cerium ions also presented excellent anti-inflammation. It could be demonstrated that the level of inflammation was related with wound repair results, indicating that excellent antibacterial impact was beneficial to the anti-inflammation and promoting the infected wound repair.

It has been demonstrated that angiogenesis takes part in wound healing process. Therefore, the impacts of the FEP and FEPC hydrogel on pro-angiogenesis were evaluated by VEGF and CD31 staining. The large number of CD31 signals were observed in the FEPC hydrogel group, while CD31 was downregulated in the 3M and FEP groups, showing fewer CD31 signals (Figure 6E and F). Meanwhile, the VEGF signals was largest in the FEPC group (Figure 6G and H). The increased CD 31 and VEGF expression in the wounds treated with FEPC hydrogel was dominated by the pronounced angiogenic behavior of the Ce^3+^, released from the FEPC hydrogel. Taken together, the FEPC hydrogel could significantly promote the infected wound repair through eradicating bacteria, reducing inflammation and improving angiogenesis.

## Discussion

Here, a novel FEPC hydrogel was constructed using Ce^3+^ and FE to cross-link with γ-PGA through double dynamic electrostatic interaction and coordination, which had outstanding injectable, self-healing, antioxidant, antibacterial features, anti-inflammation and vascularization for enhanced MRSA infected wound repair. The FEPC hydrogel achieved outstanding injectable and self-healing features owing to the dynamic coordination and electrostatic interactions. Importantly, FEPC hydrogel showed strong antioxidant property and effectively eliminated reaction oxygen species (ROS), which was because γ-PGA possessed good antioxidant performance [28], which endowed the FEPC hydrogel with excellent free radical scavenging capacity. In addition, the FEPC hydrogel could also suppress the expression of pro-inflammatory cytokines *IL-6* and *TNF-α*, presenting excellent anti-inflammatory properties. This reason for the result was that the FEPC hydrogel could effectively scavenge ROS, thereby reducing inflammation reaction, which could eliminate the overproduction inflammation in the infected wounds. Furthermore, FEPC hydrogel could promote endothelial cell migration and vascular related cytokine *VEGF* expression, which was because Ce^3+^ could enhance cell migration, a key step of angiogenesis. Meanwhile, the Ce^3+^ could also stimulate angiogenesis through stabilizing HIF-1α in the endothelial cells [35]. Angiogenesis was conducive to wound repair, which could offer the nutrients to the wound sites. Therefore, these results illustrated that FEPC hydrogel could effectively promote angiogenesis that was beneficial for wound repair. At last, the full-thickness MRSA infected derma wound model *in vivo* confirmed that FEPC hydrogel could availably eradicate bacterial infections as well as promote wound repair and tissue regeneration. During the wound repair process, the inflammation related factors were downregulated and angiogenesis related factors were upregulated.

Compared with previous studies on the other dynamic hydrogel for infected skin wound repair, the FEPC hydrogel showed several merits. First, FEPC hydrogel possessed remarkable antioxidant and anti-inflammation behaviors, which could effectively reduce excessive oxidative stress and inflammatory environment in the infected wound. Second, FEPC hydrogel could promote vascularization, a vital step during the wound repair, which offered nutrition and oxygen to the wound beds. Lastly, the FEPC hydrogel could dramatically speed up the infected wound repair time. After treated with FEPC hydrogel for Day 3, the wound closure rate was about 54.6%, which was significantly higher than previously reported hydrogel in MRSA infected wound repair on Day 3 (Figure 4H). Collectively, this study developed a neoteric antibacterial hydrogel for effectively enhancing infected wound repair.

## Conclusions

In this work, a multifunctional bioactive cerium-polypeptide hybrid dynamic FEPC hydrogel was developed through double dynamic electrostatic interaction and coordination for enhanced MRSA infected wound repair. The injectable and self-healing performance of FEPC hydrogel could be facilely *via* dynamic electrostatic interaction and coordination strategy, which made it adapt the irregular wounds. The FEPC hydrogel could effectively scavenge ROS, showing strong antioxidant property, while possessing excellent biocompatibility. The antioxidant FEPC hydrogel could significantly decrease the intracellular oxidation stress and inflammation factor, promote endothelial cell migration and angiogenesis. FEPC hydrogel could also efficiently kill *E. coli*, *S. aureus* and MRSA bacteria, eradicate MRSA bacterial infections *in vivo*, reduce inflammation, promote angiogenesis and wound repair. This study suggested that bioactive metal ions crosslinked polypeptide hydrogel should be a promising bioactive biomaterial for MDR bacteria infected wound repair.

## Funding

This work was jointly supported by the Postdoctoral Fellowship Program of China Postdoctoral Science Foundation (Grant No. GZC20232041), China Postdoctroral Science Foundation (Grant No. 2024M752538), the Fundamental Research Funds for the Central Universities (Grant No. XJSJ24081), Xidian University Specially Funded Project for Interdisciplinary Exploration (Grant No. TZJH2024024), the Key R&D Plan of Shaanxi Province of China (Grant No. 2021GXLH-Z-052) and Young Talent Support Plan of Xi’an Jiaotong University of China (Grant No. QY6J003).

## Supplementary Material

rbaf071_Supplementary_Data
