# Supplementary material for: Multifunctional dynamic cerium-polypeptide hydrogel with antibacterial antioxidative anti-inflammatory for multidrug-resistant bacterial infected wound healing
Source: Regen Biomater. 2025 Jul 1;12:rbaf071. doi: 10.1093/rb/rbaf071 (PMC12313022; doi:10.1093/rb/rbaf071)
Supplement: rbaf071_Supplementary_Data [file rbaf071_supplementary_data.zip › FEPC Manuscript- SI-revised.docx]

**Supporting Information**

**Multifunctional Dynamic Cerium-Polypeptide Hydrogel with Antibacterial Antioxidative Antiinflammatory for Multidrug-Resistant Bacterial Infected Wound Healing**

Meng Luo^a, b^*, Jing Tian^b^, Chenxi Xie^b^, Yanzi Zhao^b^, Bo Lei^b^

*^a^ School of Life Science and Technology, Xidian University, Xi’an 710126, China*

*^b^ Frontier Institute of Science and Technology, Xi’an Jiaotong University, Xi’an 710054, PR China*

* To whom correspondence should be directed

E-mail: luomeng@xidian.edu.cn

1. **Materials**

Pluronic F127 (F127), p-Toluenesulfonyl chloride (TsCl) were purchased from SigmaAldrich (St. Louis, MO, USA. ε-polylysine (EPL) (Mw 3.5 kDa) were obtained from Nanjing Shineking Biotechnology. Poly-γ-glutamic acid (γ-PGA) and cerium chloride (CeCl_3_) were offered by Alfa Aesar. Trimethylamine, dimethyl formamide (DMF) and ether were provided by J&K Scientific. ABTS assay kit was purchased from Beyotime. Phosphate buffered saline (PBS), Dulbecco’s Modified Eagle Medium (DMEM), and Alamar Blue kit were supplied from Invitrogen. IL-6 antibody and TNF-α antibody were purchased from Abcam. VEGF antibody was purchased from Cell Signaling.

1. **Synthesis of F127-EPL (FE) polymer**

In order to prepare FE polymer, the intermediate of F127-TsCl should be synthesized firstly. In brief, 0.5 mmol F127 was dispersed in 80 mL dichloromethane and 5 mmol triethylamine was added after completely dissolved. Then 5 mmol p-toluenesulfonyl chloride was dissolved in dichloromethane and slowly dropped into the above system under the nitrogen environment. The mixture system was reacted at room temperature for 3 days. After the reaction, a certain amount of hydrochloric acid and saturated sodium bicarbonate were added respectively to remove the impurities. Finally, the product was precipitated with ether and dried to obtain the F127-TsCl intermediates. FE polymers were synthesized as follows. 0.5 mmol F127-TsCl and 1 mmol EPL were added in 50 mL DMF solution and reacted at 60℃ for 3 days. The as-prepared FE polymer was further purified by dialyzed for 3 days and finally lyophilized.

1. **Characterizations**

The nuclear magnetic resonance (^1^H NMR) (NICOLET 6700, Thermal) and fourier transform infrared (FT-IR) spectroscopy (Ascend 400 MHz, Bruker) were employed to characterize the physicochemical structure of the FE and FEPC hydrogel. The scanning electron microscopy (SEM) (GeminiSEM 500, Zeiss) was used to observe the morphology of the hydrogels. The composition of the FEPC hydrogel was analyzed by energy dispersive spectroscopy (EDS) attached to the SEM.

1. **Rheological assessment**

The TA rheometer (DHR-2) was used to measure the rheological performance of FEPC hydrogel. Briefly, the storage modulus (G') and loss modulus (G'') of hydrogels were measured under room temperature at 1 % oscillation strain and 1 Hz frequency. The viscosity of hydrogels was tested under room temperature and shear rate was changed from 0.1 to 100 1/s. The time sweeps were used to indicate the change of G′ and G″ which were recorded by time sweeps with fixed temperature (37°C) and frequency (1 Hz). The shear-thinning behavior of the hydrogels was carried out by changing the strain (from 1% to 1000% with 100 s interval) for three cycles.

1. **Self-healing and injectability evaluation**

To evaluate the self-healing capability of the hydrogel, 500 μL hydrogel was put into a small plate. Then a little crack was made in the middle of the hydrogel. The self-healing behavior of the hydrogel was observed by the microscopy at regular time points. The injectability was test by the syringe. The 800 μL hydrogel was placed in a 1 mL syringe and extruded it out without resistance, writing a “FEPC” shape.

1. ***In vitro antioxidan*t efficiency assay**

The antioxidant efficiency was also evaluated through measuring the ability to scavenge the ABTS^+^ free radicals. First, a 7.4 mM ABTS solution and 2.6 mM potassium persulfate (K_2_S_2_O_8_) were mixed at room temperature in the dark for 12 h to prepare the ABTS radical cation (ABTS^+^) solution. Then, the ABTS^+^ solution was diluted using deionized water to an absorbance of 0.70 ± 0.02 at 734 nm. Then, 200 μL of the ABTS^+^ solution was added to the surface of the hydrogel and reacted in the dark for 5 min. The absorbance of the solution at 734 nm was recorded. The ABTS^+^ radicals scavenging ability was calculated as follow: ABTS^+^ radicals scavenging ability (%) = (A_0_-A_s_)/A_0_ x 100, where A_0_ was the absorbance of the ABTS^+^ liquid and A_s_ was the absorbance of the ABTS^+^ liquid-containing various hydrogels (n=3).

1. **Hemocompatibility evaluation**

The erythrocytes were collected from fresh mouse blood centrifuging (1000 rpm) of the mouse blood for 10 min and washed with sterile PBS (pH = 7.4) five-six times *via* centrifugation. The purified erythrocytes were further diluted 20 times with PBS to obtain the stock dispersion. After that, the erythrocytes were incubated with the hydrogels in 24-well culture plate. After placed at 37 °C for 1 h with a shaking speed of 100 rpm, the mixtures were centrifuged to remove hemolysis-free erythrocytes. The supernatants (80 µL) were carefully transferred into a new 96-well clear plate to read the absorbance of the solutions at 540 nm with a microplate reader (SpectraMax@, Molecular Services). PBS and TritonX-100 were served as the controls (negative and positive, respectively). The hemolysis rate was calculated as follow: Hemolysis rate = [ (absorbance of sample -absorbance of negative control) / (absorbance of positive control -absorbance of negative control)] x 100%, (n=3).

1. **Cytotoxicity evaluation**

The cytotoxicity of the hydrogels was carried out according to our previous work using fibroblast cells (L929), human umbilical vein endothelial cells (HUVECs), and RAW264.7. Briefly, the cells were cultured in Dulbecco’s modified Eagle’s medium (DMEM, Invitrogen, USA) with 10% fetal bovine serum (FBS) and 1% peniciline-streptomycin at 37℃ with 5% CO_2_. The cells were seeded into 96-well plate with the density of 3000 cells/well. The extract of the hydrogels was placed into the well after the cells adhere to the plate. On day 1 and day 3, the cell viability of the hydrogels was test by an Alamar Blue kit.

1. **Cell migration evaluation**

The effect of the FEPC hydrogel on cell migration was estimated using HUVECs. In brief, HUVECs (1x10^5^ cell per well) were firstly seeded into the 12-well plates and cultured until adherence with 10% FBS medium. The cell scratch wounds were created mechanically by a 10 μL tip, and washed with PBS for twice. Then the medium was replaced by the extract of the hydrogels with 0% FBS medium. Finally, the scratch wound was monitored and analyzed at regular intervals. The relative healing area (%) was calculated as follow: Relative healing area (%)= (A_0_-A_t_)/A_0_ × 100, where A_0_ was the original scratch wound area and A_t_ was the scratch wound area at the regular time intervals (n=3).

1. **Pro-angiogenesis evaluation**

The HUVECs were seeded into the 12-well plate (1x10^5^ cell per well). After the cells were adhered to the plate, the hydrogel extract was added into each well and culture for 48 h. The RNA expression level of angiogenesis-related genes VEGF was detected by real-time reverse transcriptase-polymerase chain reaction (RT-PCR). In brief, the total RNA was extracted using Trizol reagent. Subsequently, the obtained RNA was reverse-transcribed into cDNA. Finally, the angiogenesis-related genes VEGF was evaluated.

1. **Anti-inflammation evaluation**

Anti-inflammation evaluation: The RAW264.7 cells were seed into the 12-well plate (1x10^5^ cell per well) and cultured for adhered plate. Then, the cells were treated with LPS (100 ng mL-1) for 12 h. Next, the cell medium was replaced by hydrogel extract for 48 h. Lastly, the levels of the pro-inflammatory genes including IL-6 and TNF-α were evaluated. The detailed experimental procedure was similar to that of angiogenesis.

**12. *In Vitro* Antibacterial Assays**

*E. coli* (non-MDR Gram-negative bacteria, non-MDRGNB), *S. aureus* (non-MDR Gram-positive bacteria, non-MDRGNP) and MRSA (methicillin-resistant Staphylococcus aureus, multidrug resistant bacteria, MDR) were used to estimate the antibacterial performance of FEPC hydrogels *in vitro*. In brief, the 400 μL hydrogels were putted in a 24-well plate. Subsequently, 10 μL bacterial suspension (10^6^ CFU mL^-1^ in PBS) was dropped onto the surface of the above hydrogel surface and 990 μL PBS which was used as a control. Then the plate was incubated at 37 ℃ for 2 h. The 990 μL PBS was added into the hydrogel containing bacterial to ensure the bacterial resuspended. Subsequently, 10 μL bacterial suspension was coated evenly on LB agar and cultured at 37 ℃ for 12-16 h. The alive bacteria were counted by colony forming units on agar plate. The bactericidal rate was calculated as follows: Kill (%) = (N_c_-N_s_) / N_c_×100%, N_s_ and N_c_ is the number of bacteria in the hydrogel group and control group, respectively.

**13. *In vivo* infected wound healing evaluation**

An infectious skin defect model was established to evaluate the effects of FEPC hydrogel according our previous work. In brief, the female mice (30-35 g) were randomly divided into three cohorts (n=6): 3M (the commercial dressing), FEP hydrogel, and FEPC hydrogel. The round wound (diameter=8 mm) was created in the dorsal of the mice after anesthetized. The 10 μL bacterial suspension of MRSA (n × 10^7^ CFU/mL) was dropwise onto the wound beds and treated with various hydrogels. The wound area was observed and photographed on day 0, 3, 7, 14. The wound contraction (%) was calculated as follow: Wound contraction (%) = (A_0_-A_t_)/A_0_ × 100, where A_0_ was the original wound area and A_t_ was the wound area at the regular time intervals. For histological analysis, the wound tissues were collected and fixed 4% paraformaldehyde. The Hematoxylin-Eosin (H&E) staining, Masson’s trichrome-staining, and immunohistochemical staining of IL-6, TNF-α, and VEGF were employed to analyze the inflammatory response and angiogenic at the wound beds. H&E, Masson's trichrome sections and immunofluorescence sections were observed with an upright fluorescence microscope (BX53, Olympus) and an inverted fluorescence microscope, respectively (IX53, Olympus). Image J software was applied to determine quantification of microvessels and the proportion of collagen deposition by measuring the intensity of the blue areas. In addition, an Image J software was also used to analyze the wound size and the semi-quantitative expression of IL-6, TNF-α and VEGF.


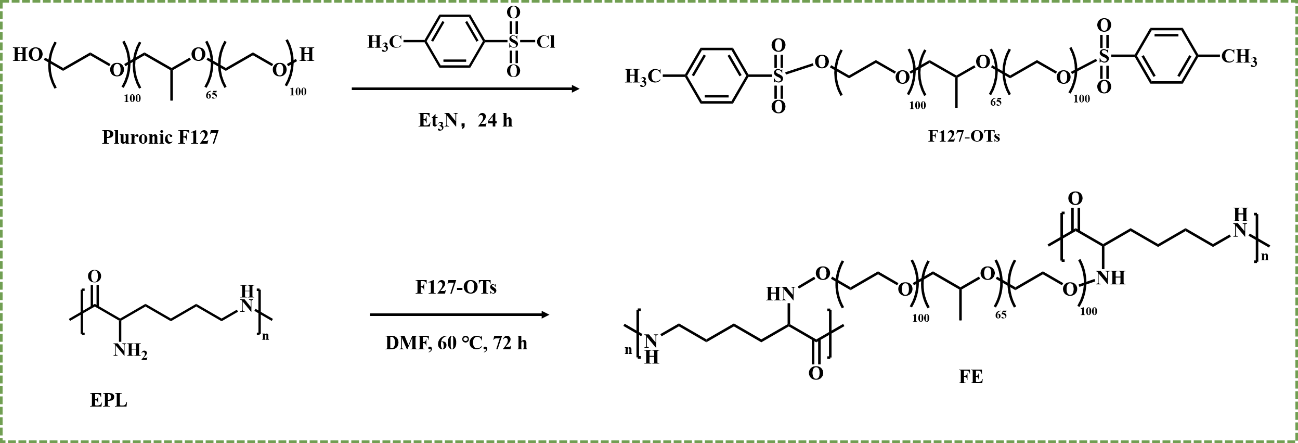


**Figure S1. The synthetic procedure of the FE polymer.**


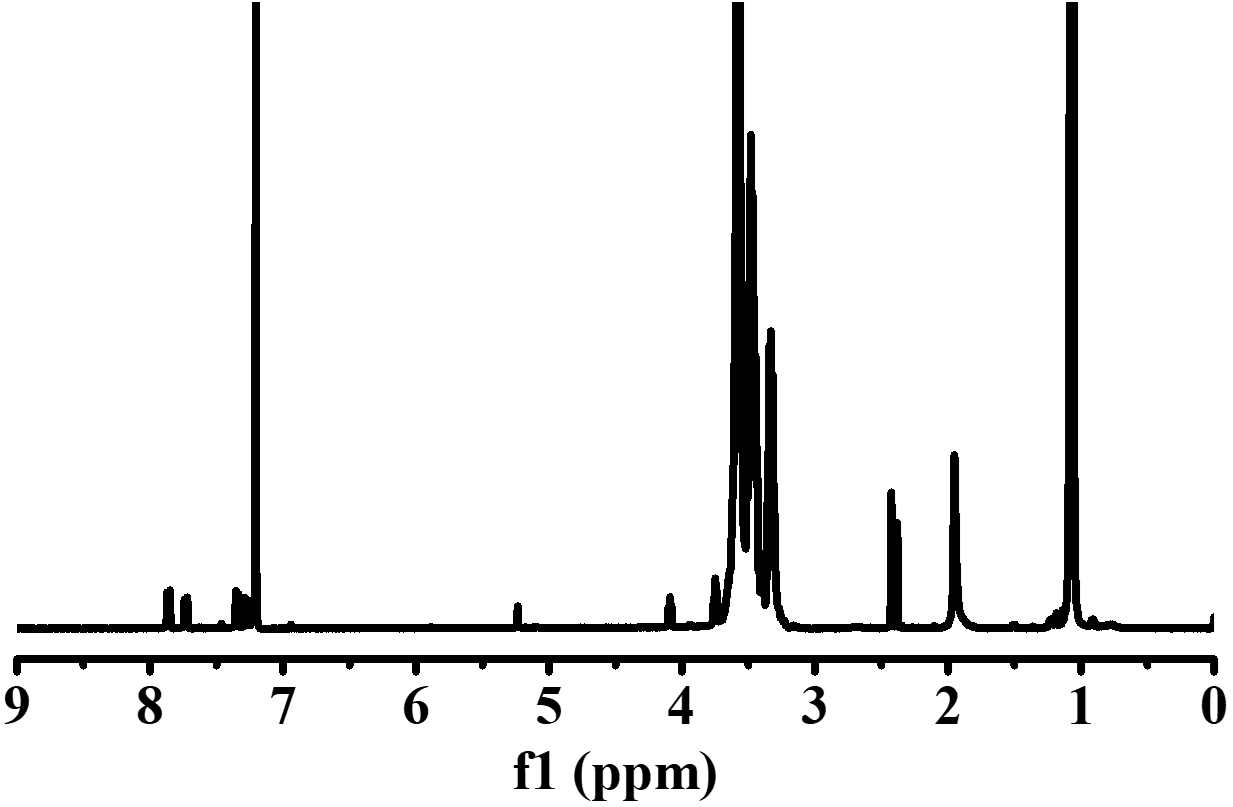


**Figure S2. The ^1^H NMR of F127-TsCl intermediate.**


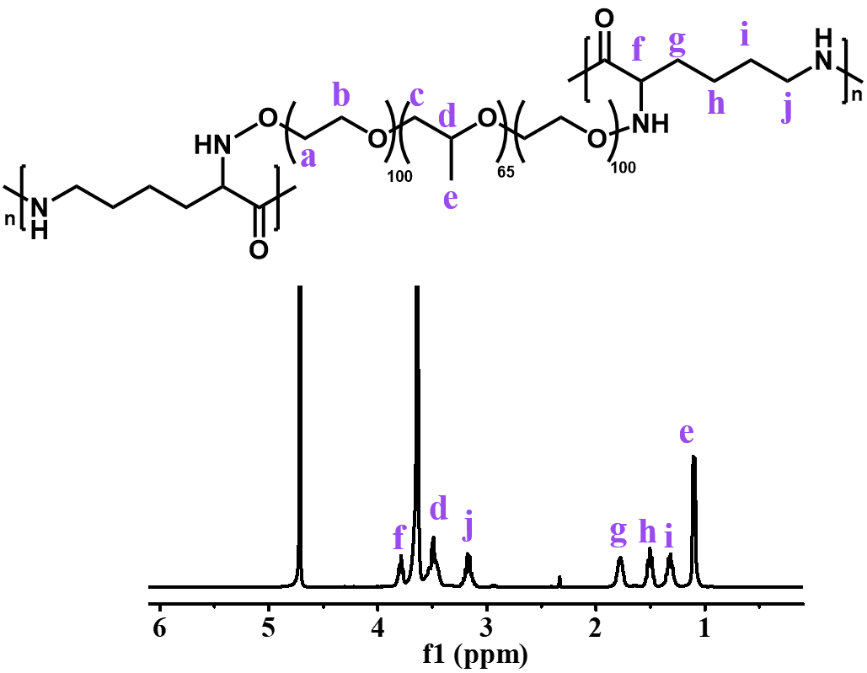


**Figure S3. The ^1^H NMR of FE polymer.**


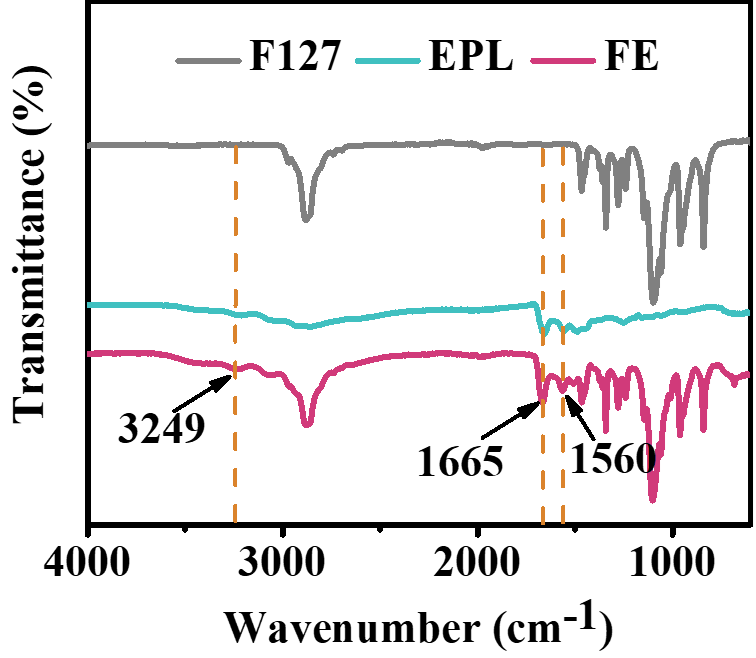


**Figure S4. The FT-IR spectra of FE polymer.**


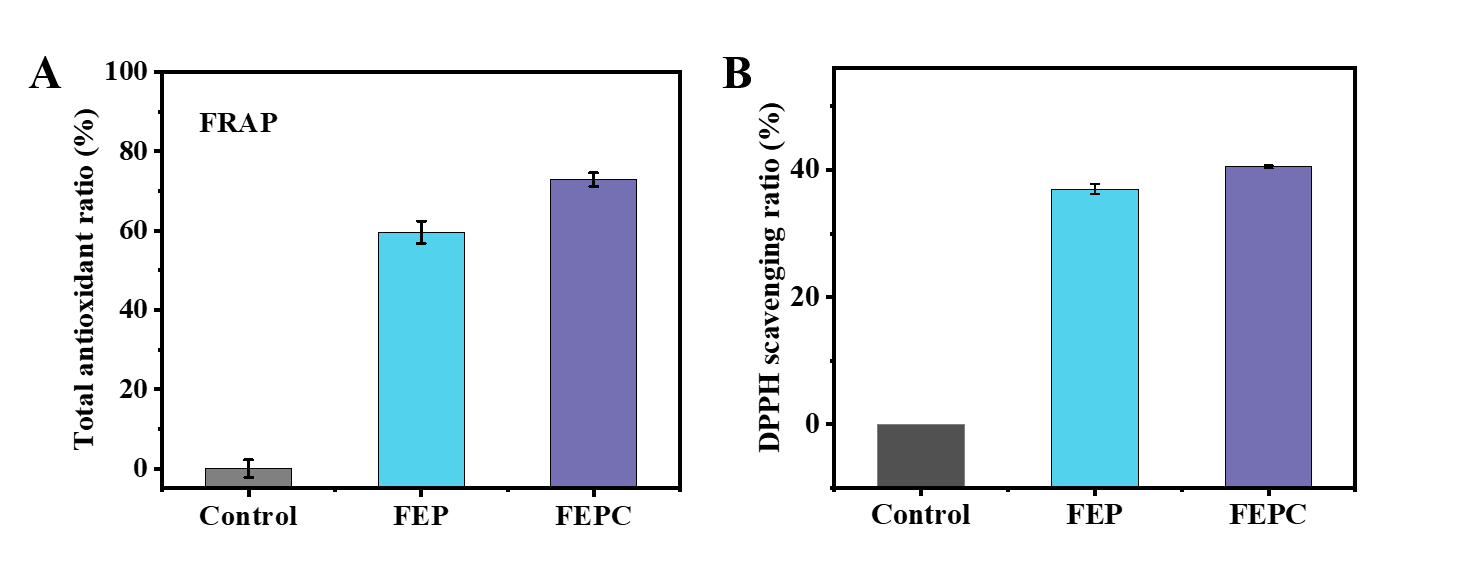


**Figure S5. Antioxidant evaluation of hydrogels. A) FRAP assay; B) DPPH scavenging ratio.**


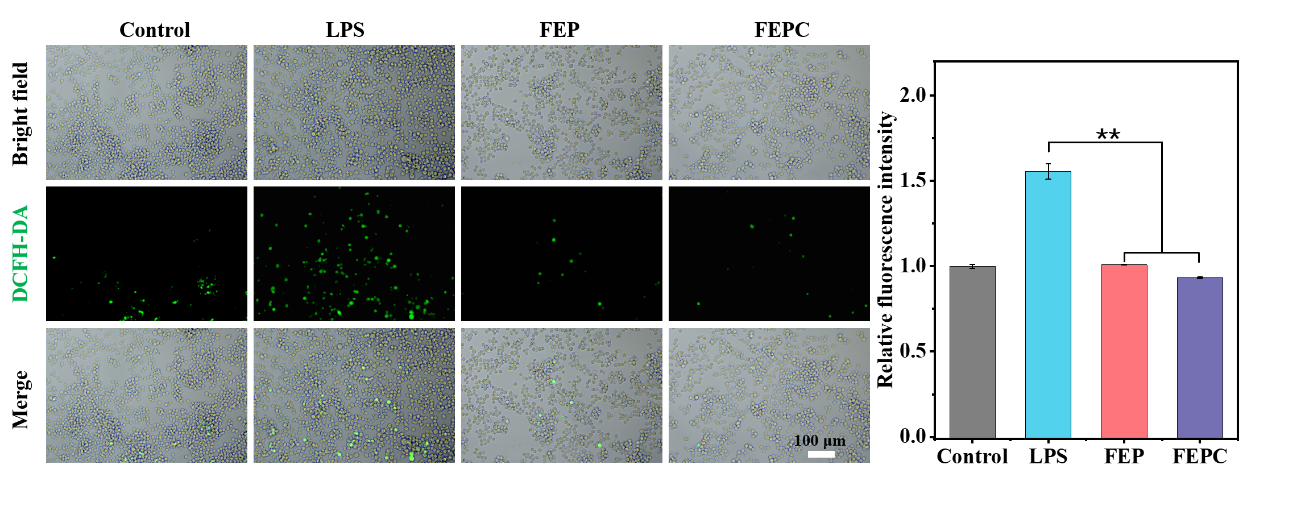


**Figure S6. Intracellular ROS scavenging ability evaluation.**


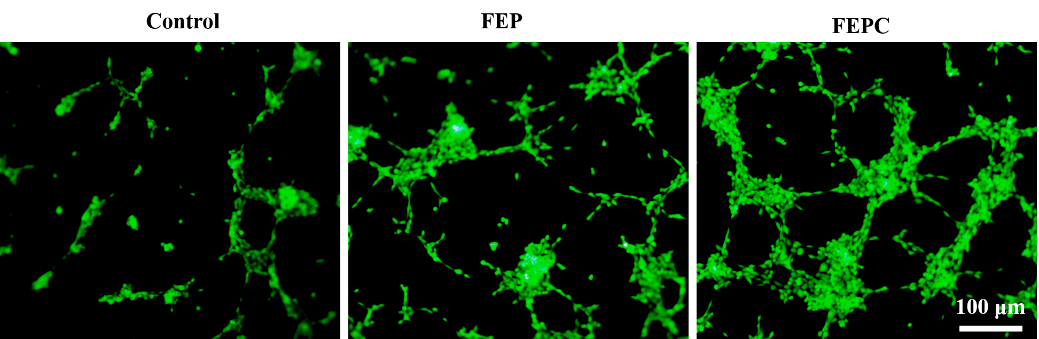


**Figure S7. Tubule formation of HUVECs *in vitro*.**


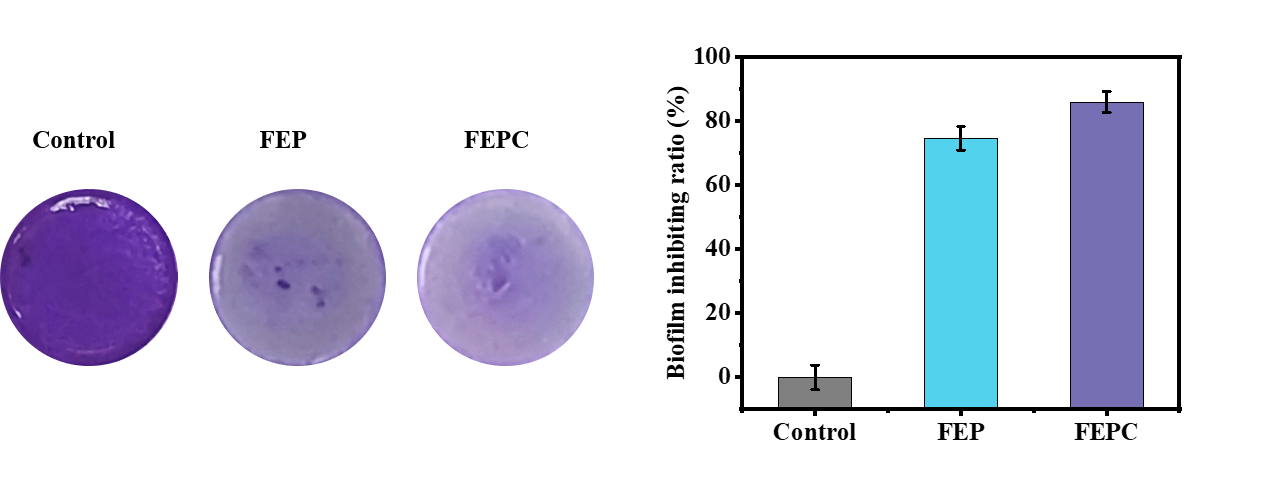


**Figure S8. Evaluation of anti-biofilm performance *in vitro*.**

**
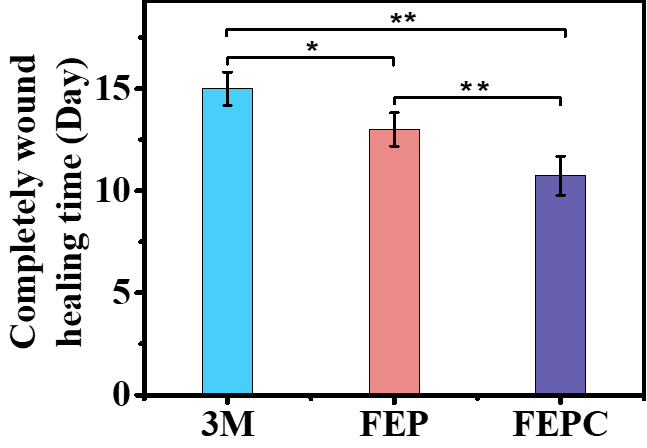
**

**Figure S9. Complete wound healing days after treatment of various samples.**


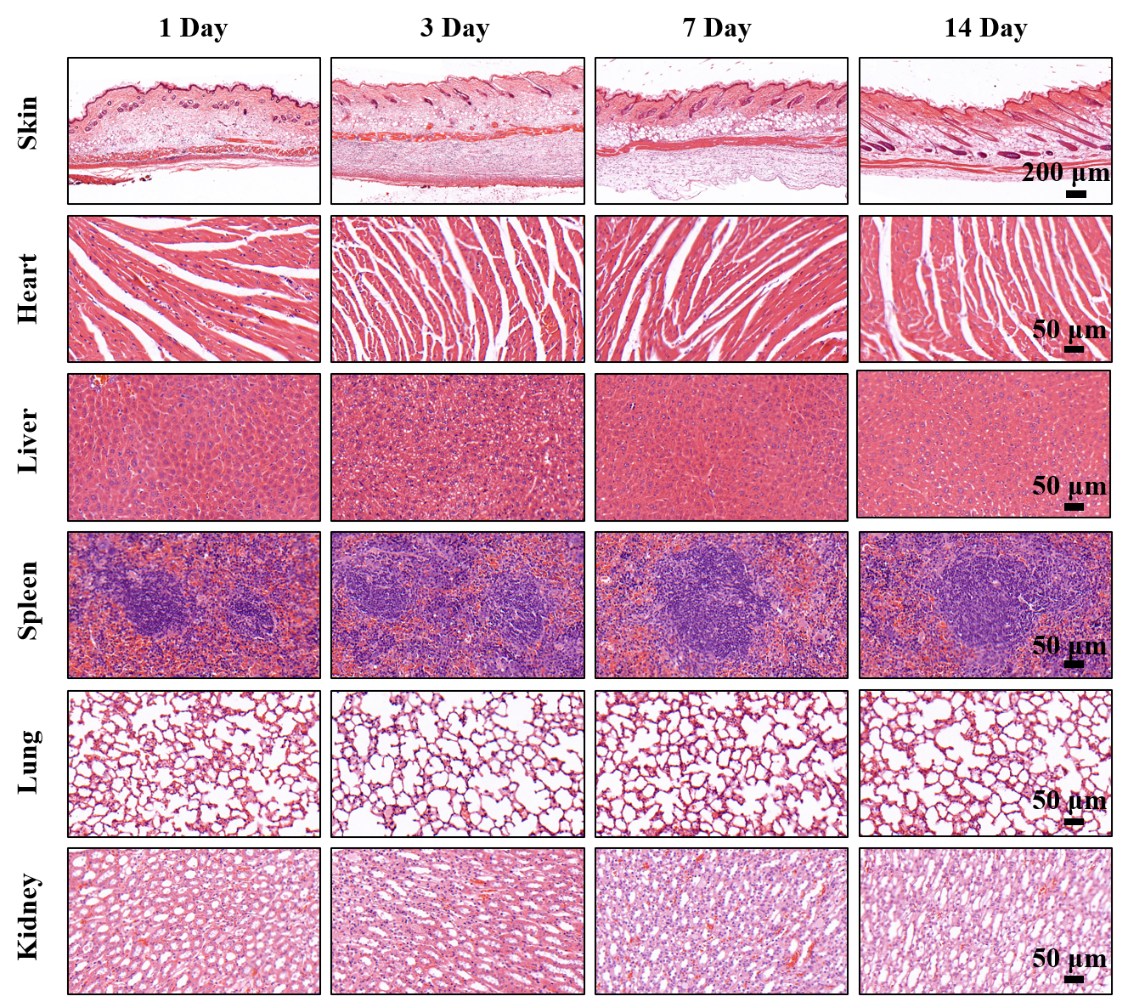


**Figure S10. H&E staining of tissue sections at specific time point.**
